# Muscle Histidine-Containing Dipeptides Are Elevated by Glucose Intolerance in Both Rodents and Men

**DOI:** 10.1371/journal.pone.0121062

**Published:** 2015-03-24

**Authors:** Sanne Stegen, Inge Everaert, Louise Deldicque, Silvia Vallova, Barbora de Courten, Barbara Ukropcova, Jozef Ukropec, Wim Derave

**Affiliations:** 1 Department of Movement and Sport Sciences, Ghent University, Ghent, Belgium; 2 Department of Kinesiology, Exercise Physiology Research Centre, KU Leuven, Heverlee, Belgium; 3 Department of Pathophysiology, Faculty of Medicine, Comenius University, Bratislava, Slovakia; 4 Monash Centre for Health, Research and Implementation, Faculty of Medicine, Nursing & Health Sciences, Melbourne, Australia; 5 Institute of Experimental Endocrinology, Slovak Academy of Sciences, Bratislava, Slovakia; Leiden University Medical Center, NETHERLANDS

## Abstract

**Objective:**

Muscle carnosine and its methylated form anserine are histidine-containing dipeptides. Both dipeptides have the ability to quench reactive carbonyl species and previous studies have shown that endogenous tissue levels are decreased in chronic diseases, such as diabetes.

**Design and Methods:**

*Rodent study:* Skeletal muscles of rats and mice were collected from 4 different diet-intervention studies, aiming to induce various degrees of glucose intolerance: 45% high-fat feeding (male rats), 60% high-fat feeding (male rats), cafeteria feeding (male rats), 70% high-fat feeding (female mice). Body weight, glucose-tolerance and muscle histidine-containing dipeptides were assessed. *Human study:* Muscle biopsies were taken from m. vastus lateralis in 35 males (9 lean, 8 obese, 9 prediabetic and 9 newly diagnosed type 2 diabetic patients) and muscle carnosine and gene expression of muscle fiber type markers were measured.

**Results:**

Diet interventions *in rodents* (cafeteria and 70% high-fat feeding) induced increases in body weight, glucose intolerance and levels of histidine-containing dipeptides in muscle. *In humans*, obese, prediabetic and diabetic men had increased muscle carnosine content compared to the lean (+21% (p>0.1), +30% (p<0.05) and +39% (p<0.05), respectively). The gene expression of fast-oxidative type 2A myosin heavy chain was increased in the prediabetic (1.8-fold, p<0.05) and tended to increase in the diabetic men (1.6-fold, p = 0.07), compared to healthy lean subjects.

**Conclusion:**

Muscle histidine-containing dipeptides increases with progressive glucose intolerance, in male individuals (cross-sectional). In addition, high-fat diet-induced glucose intolerance was associated with increased muscle histidine-containing dipeptides in female mice (interventional). Increased muscle carnosine content might reflect fiber type composition and/or act as a compensatory mechanism aimed at preventing cell damage in states of impaired glucose tolerance.

## Introduction

Histidine-containing dipeptides (HCD) are peptides consisting of a histidine (or a methylated form of histidine) and the atypical amino acid beta-alanine [1]. HCD are predominantly and abundantly present in skeletal muscle, but they are also measurable in other tissues such as brain, kidney and liver, although in concentrations 10- to 1000-fold lower [1–4]. Human skeletal muscles only possess the HCD carnosine (beta-alanyl-L-histidine), whereas rodent muscles contain carnosine along with its methylated variant anserine (beta-alanyl-N-π-methylhistidine) [2].

Carnosine supplementation has recently been associated with a delayed progression of type 1 and 2 diabetes in rodents [5–8]. The mechanism has been attributed to its biochemical property to quench and scavenge damaging species such as reactive oxygen species [9], metal-ions [10], protein carbonyls [11] and reactive carbonyl species (RCS) such as 4-hydroxy-2-nonenal (HNE) [12] and acrolein [13]. Aldini et al. (2011) was the first to report that obese Zucker rats have higher levels of carnosine-HNE adducts in urine compared to their lean counterparts, which points to HNE overproduction in the obese animals and confirms the role of carnosine as an endogenous detoxifying agent of RCS such as HNE [14]. In addition, Baba et al. (2013) confirmed the presence of carnosine-RCS conjugates (HNE and acrolein) in urine from normal, healthy, non-smoking adults and also demonstrated its existence in skeletal muscle of C57BL/6 mice [15]. Interestingly, anserine has similar characteristics as carnosine [2], although less extensively investigated.

Because of these scavenging characteristics, it is suggested that carnosine levels in diabetic tissues are decreased, as it has been shown that diabetic tissues have high production of reactive oxygen and carbonyl species [16]. Indeed, decreased carnosine and anserine levels are demonstrated in retina, kidney and liver of obese and diabetic (type 1 and 2) rodents [4,6,7,17]. However, for skeletal muscle, contradiction exists. In rodents, there is only one paper available which shows decreased carnosine levels in diaphragm muscle of STZ-induced diabetic rats [18]. In humans, Gualano et al. [19] reported reduced carnosine content (-45%) in gastrocnemius muscle in type 2 diabetic patients, but not in soleus muscle nor in type 1 diabetic patients, compared to control subjects matched for age, BMI, gender and diet. However, another study [20] demonstrated an increase in carnosine content in soleus muscle in drug naive type 2 diabetic patients compared to non-diabetic controls.

Although evidence is scarce, a new research topic about supplementing carnosine in humans to combat age related chronic diseases i.e. diabetes and cardiovascular diseases is emerging because of its physiological and scavenging properties. Beta-alanine may be used as an alternative [19,21,22], because it is the rate-limiting precursor for carnosine synthesis in muscle [23]. However, given the conflicting findings in literature concerning muscle carnosine content in a diabetic state, we investigated whether muscle carnosine content is indeed decreased in a diabetic state. We investigated this by feeding rodents various degrees of high-fat diets inducing various degrees of glucose intolerance (interventional study) and by comparing muscle carnosine content in humans with different degrees in glucose tolerance (cross-sectional study). Moreover, as it is known that exercise can reverse metabolic stress in skeletal muscle [24,25], the additional effect of daily endurance training (rodent study) or physical activity (human study), will be examined.

## Materials and Methods

### Study Design

**Rodent study (intervention study).** Skeletal muscles of rats and mice were collected from 4 different diet-intervention studies, aiming to induce various degrees of glucose intolerance. The specifications of the animals and the diets are presented in Table 1. Shortly, rodents were fed with a 45% high-fat diet, a 60% high-fat diet, a cafeteria diet or a 70% high-fat diet, and they were compared with their own control group (receiving a control diet). In the HF 70%study, an additional group was added to assess the effect of exercise, namely HF 70% diet in combination with endurance training (12 m/min for 60 min per day, 5 days a week during 6 weeks). All rodents were housed at 22°C in a 12 h light/dark cycle and were given *ad libitum* access to their water and specific diet.

**Table 1:** *Rodent study*: Specifications of the animals and their diets.

						Energy content of the diet (% total kJ)		
Study	Species	Strain	Gender	Age at sacrifice	Intervention period	Macro-nutrients	CON	HF/CAF
Rat studies								
HF 45%	Rats	Wistar	M	18 weeks	14 weeks	Total CHO	70	36
						Total fat	10	45 (mainly lard)
						Total protein	20	19
HF 60%	Rats	Sprague-Dawley	M	12 weeks	8 weeks	Total CHO	70	20
						Total fat	10	60 (mainly lard)
						Total protein	20	20
CAF	Rats	Wistar	M	18 weeks	12 weeks	Total CHO	63	69 (mainly sugar)
						Total fat	12	16
						Total protein	25	15
Mouse study								
HF 70%	Mice	C57BL/6J	F	15 weeks	6 weeks	Total CHO	Standard chow	<1
						Total fat		72 (lard + corn oil)
						Total protein		28

Abbreviations: M: male, F: female, CHO: carbohydrates, CON: control group, HF: high-fat group, CAF: cafeteria group.

In all rodent studies, body weight was recorded during the intervention study and glucose intolerance was measured at the end of the intervention (cfr infra for the description of the glucose tolerance tests). Thereafter, all animals were sacrificed by an intra-peritoneal injection of sodium pentobarbital solution and muscles were dissected for the determination of muscle HCD (carnosine and anserine). In the rat studies, the white gastrocnemius (GW) and the red gastrocnemius (GR) were dissected because of a different muscle fiber type distribution [26]. In the mouse study, the gastrocnemius was taken as a whole (GAS), because it is difficult to make a visual distinction between the GR and GW in mice. The muscle samples were frozen in liquid nitrogen and stored at −80°C until HCD content analysis. All rodent studies were approved by the local animal Ethical Committee (the rat studies by KU Leuven: permit number P037/2010, the mouse study by Université catholique de Louvain: permit number LA 1220548). All surgery was performed under anesthesia (cfr. Infra) and all efforts were made to minimize suffering.

**Human study (cross-sectional study).** The study population consisted of 35 sedentary non-vegetarian males with different degrees of body weight and glucose intolerance: 9 healthy lean, 8 obese with normal glucose tolerance, 9 prediabetic (preT2D) and 9 newly diagnosed drug naïve type 2 diabetic men (T2D). Prediabetes (impaired glucose tolerance and/or impaired fasting glucose) and type 2 diabetes were diagnosed by fasting glycemia or 2 hours OGTT (oral glucose tolerance test) as defined by the American Diabetes Association 2006 [27]. Individuals were age-matched across the groups, with a mean age of 45 ± 7 yrs. The obese, preT2D and T2D were BMI-matched across the groups. The samples of m. vastus lateralis were obtained by the Bergstrom needle biopsy. Muscle samples were immediately frozen in liquid nitrogen and stored at −80°C until gene expression and carnosine content analyses. Fasting insulinemia was measured by IRMA (Immunotech, France). Daily free living ambulatory activity was monitored by accelerometers (Lifecorder Plus, Kenz, USA) during three consecutive working days. Medium and high intensity ambulatory activity was defined as an activity with the energy requirements exceeding 3-times the resting energy expenditure (>3 REE) [28].

The study was approved by the Ethics Committee of the University Hospital Bratislava, Comenius University Bratislava and the Ethics Committee of the Bratislava Region Office and it conforms to the ethical guidelines of the Helsinki declaration of 1964, as revised in 2000. All participants provided witnessed written informed consent prior entering the study.

### Glucose tolerance tests

**Rodent study.** *The intravenous glucose tolerance tests (IVGTT)* in the *rat studies* (the HF 45%, HF 60% and CAF-study) were performed according to Vaisy et al. (2011)[29]. Briefly, rats were anaesthetized by an intraperitoneal injection of a ketamine-xylazine mixture and surgically prepared for the IVGTT, which involved catheter insertion into the left jugular vein. After an overnight fast (16–18 h) a glucose solution (1g glucose.kg^-1^ body weight using a 30% w.v.^-1^ glucose solution in w.v^-1^ saline) was injected into the catheter of the conscious rats and blood glucose and plasma insulin concentrations were measured at regular intervals. Total AUC_glucose_ and AUC_insulin_ were calculated using the trapezoidal rule.

*The oral glucose tolerance tests (OGTT)* from *the mouse study* is described in Deldicque et al. (2013) [30]. Plasma glucose was determined on regular intervals following oral glucose gavage (glucose 1 mg/g body weight), plasma insulin was only determined 30 min before and 15min after glucose gavage. Total AUC for glucose (AUC_glucose_) was calculated using the trapezoidal rule.

### Quantification of muscle carnosine, anserine and total HCD

HCD (carnosine and anserine) were quantified by means of reversed-phase HPLC (high-performance liquid chromatography). Skeletal muscles were dissolved in phosphate buffer (rat studies: 1mg dw muscle/100μL PBS; mouse study: 10mg ww muscle/100 μLPBS; human study: 1mg ww muscle/15 μL PBS) for homogenization. Muscle homogenates were deproteinized using 35% sulfosalicylic acid (SSA) and centrifuged (5min, 14000g). 100μL of deproteinized supernatant was dried under vacuum (40°C). Dried residues were resolved with 40μL of coupling reagent: methanol/triethylamine/H_2_O/phenylisothiocyanate (PITC) (7/1/1/1) and allowed to react for 20 minutes at room temperature. The samples were dried again and resolved in 100μL of sodium acetate buffer (10mM, pH 6.4). The same method was applied to the combined standard solutions of carnosine (Flamma) and anserine (Sigma). The derivatized samples (20 μL) were chromatographed on a Waters HPLC system with ODS2 guard column (80Å, 5 μm, 4.6 mm X 10 mm), a Spherisorb C18/ODS2 column (4.6 x 150 mm, 5 μm), and UV detector (wavelength: 210 nm). The columns were equilibrated with buffer A (10 mM sodium acetate adjusted to pH 6.4 with 6% acetic acid) and buffer B (60% acetonitrile-40% buffer A) at a flow rate of 0.8 ml/min at 25°C. The limit of quantification of muscle homogenates was 7 μM (~0,7 mmol/kg DW muscle). Total HCD are the sum of muscle carnosine and anserine.

### The gene expression analysis

Gene expression analysis of muscle fiber type markers MYH1 (encoding the protein MyHC ‘myosin heavy chain’ 2X, present in fast-glycolytic muscle fibers), MYH2 (encoding the protein MyHC 2A, present in fast-oxidative muscle fibers) and MYH7 (encoding the protein MyHC-β/slow, present in slow-oxidative muscle fibers) was performed in *m*. *vastus lateralis*. Total RNA from skeletal muscle was isolated using TriReagent (Molecular Research Center, Inc., USA), purified with RNeasy mini Kit (Qiagen, USA) and DNAse treated (Qiagen, USA). Gene expression was measured by the qRT-PCR with ABI7900HT (Applied Biosystems, USA), using following set of primers designed with the PrimerExpress software (Applied Biosystems, USA): MYH1 (FWD: 5´- TAA GAC CGA GGC AAA AAG GA-3´ REV: 5´- TGC ATC AGC CAA GCT GTC-3´); MYH2 (FWD: 5´-TGT CTC ACT CCC AGG CTA CA-3´ REV: 5´- CCA AAA ACA GCC AAT TCT GAG-3´); MYH7 (FWD: 5´-CTT CGT GCC TGA TGA CAA ACA-3´ REV: 5´- CAC GGT CAC TGT CTT GCC ATA-3´) and Maxima SYBRgreen/ROX gPCR master mix (Fermentas Thermo Scientific, USA). B-2-microglobulin (B2m), Ribosomal protein L13a (Rpl13a) & Hypoxanthine phosphoribosyltransferase 1 (HPRT1) were stably expressed across the experimental groups and therefore used as the set of internal reference genes to calculate dCt expression values. HPRT1 was determined with the aid of Taq-man gene expression assay (Hs03929098_m1, Applied Biosystems, USA) while *B2m* (fwd: CGCTCCGTGGCCTTAGC; rev: AATCTTTGGAGTACGCTGGATAGC) and *Rpl13a* (fwd: GGACCGTGCGAGGTATGCT; rev: ATGCCGTCAAACACCTTGAGA) with the above specified set of primers. PCR efficiency for the genes of interest and reference genes was tested and optimized before the real time PCR experiment.

### Statistics

**Rodent study.** To compare body weight, AUC_glucose_, AUC_insulin_, muscle carnosine, anserine and HCD between the HF/CAF groups (HF 45%, HF 60%, CAF or HF 70%) and their own control group, data were analyzed by an independent t-test. To compare muscle carnosine, anserine and HCD between the 3 groups (CON, HF 70% and HF 70%+ex) in the HF 70% study, one-way ANOVA was performed. When a significant group effect was shown, a post hoc LSD test was performed. The pearson correlation coefficient was calculated to explore the relationship between muscle HCD and glucose tolerance (bloodglucose during OGTT/IVGTT).

**Human study.** To compare muscle carnosine and gene expression levels between the 4 groups (lean, obese, preT2D and T2D), one-way ANOVA was performed. When a significant group effect was shown, a post hoc LSD test was done. All correlations were evaluated by Pearson correlations. All analyses (human and rodent) were done with SPSS statistical software (SPSS 20, Chicago, IL) and statistical significance was set at p < 0.05.

## Results

### Body weight and glucose tolerance

**Rodent study (Table 2)**. In the *HF 45% study*, body weight tended (p = 0.08) to be higher in HF compared to CON, but glucose tolerance (AUC_glucose_ and AUC_insulin_) was not significantly altered. In the *60% HF study*, body weight and AUC_insulin_ were higher in HF (+ 12% and + 71% respectively, p<0.05) compared to CON, and AUC_glucose_ tended to be higher (+ 22%, p = 0.054). In the *CAF study*, body weight, AUC_glucose_ and AUC_insulin_ were all higher in HF compared to CON (+ 12%, 25% and 139% respectively, p<0.05). In the *HF 70% study*, both body weight and AUC_glucose_ were higher in HF compared to CON (+ 14% and + 81% respectively, p<0.05). In exercised high-fat diet mice (HF 70%+ex), body weight was normalized back to levels of CON mice, however, AUC_glucose_ did not decrease by exercise. Plasma insulin in fasted state and at 15 min after the OGTT was not different between the three groups.

**Table 2:** *Rodent study*: Degree of obesity and glucose tolerance for the 4 diet-interventions.

Study		Obesity		Glucose tolerance test			
		Delta body weight (g)	End body weight (g)	Total AUC for glucose		Total AUC for insulin	
				mmol.l^-1^.min	% increase	ng.ml^-1^.min	% increase
Rat studies							
HF 45%	CON (n = 8)	323 ± 32	430 ± 34	999 ± 138		463 ± 134	
	HF (n = 7)	377 ± 61 *	480 ± 69 ^$^	1038 ± 127	4	533 ± 246	15
HF 60%	CON (n = 9)	334 ± 48	486 ± 57	1144 ± 186		171 ± 74	
	HF (n = 9)	390 ± 44 *	545 ± 47 *	1397 ± 305 ^$^	22	293 ± 139 *	71
CAF^a^	CON (n = 11)	255 ± 14	490 ± 47	579 ± 29		185 ± 28	
	CAF (n = 11)	305 ± 10*	548 ± 55 *	721 ± 44 *	25	443 ± 39 *	139
Mouse study							
HF 70%^a^	CON (n = 8)	3.9 ± 1.0	21.6 ± 1.9	168 ± 68			
	HF (n = 8)	6.1 ± 1.1 *	23.1 ± 1.5	304 ± 127 *	81	not available	
	HF + ex (n = 6)	4.7 ± 1.3 **	20.7 ± 1.4**	280 ± 71 *	67		

Values are expressed as mean ± SD.

* p<0.05 versus CON,

** p<0.05 HF+ex versus HF and

^$^ p<0.1 versus CON.

Underlined % increase (compared to CON) are significant with p<0.05.

^a^Data from the CAF and HF 70% study are obtained from respectively Vaisy et al. (27) and Deldicque et al.(28).

Abbreviations: CON: control group, HF: high-fat group, CAF: cafeteria group, HF+ex: high-fat+exercise group.

**Human study.** Body weight and BMI of the lean subjects was significantly lower compared to the other three groups (body weight: lean: 78±10, obese: 99±21, preT2D: 103±8, T2D: 97±12 kg; BMI: lean: 25±1, obese: 29±3, preT2D: 32±2, T2D: 31±3 kg/m^2^).

### Muscle HCD and muscle fiber type markers

**Fig 1:**
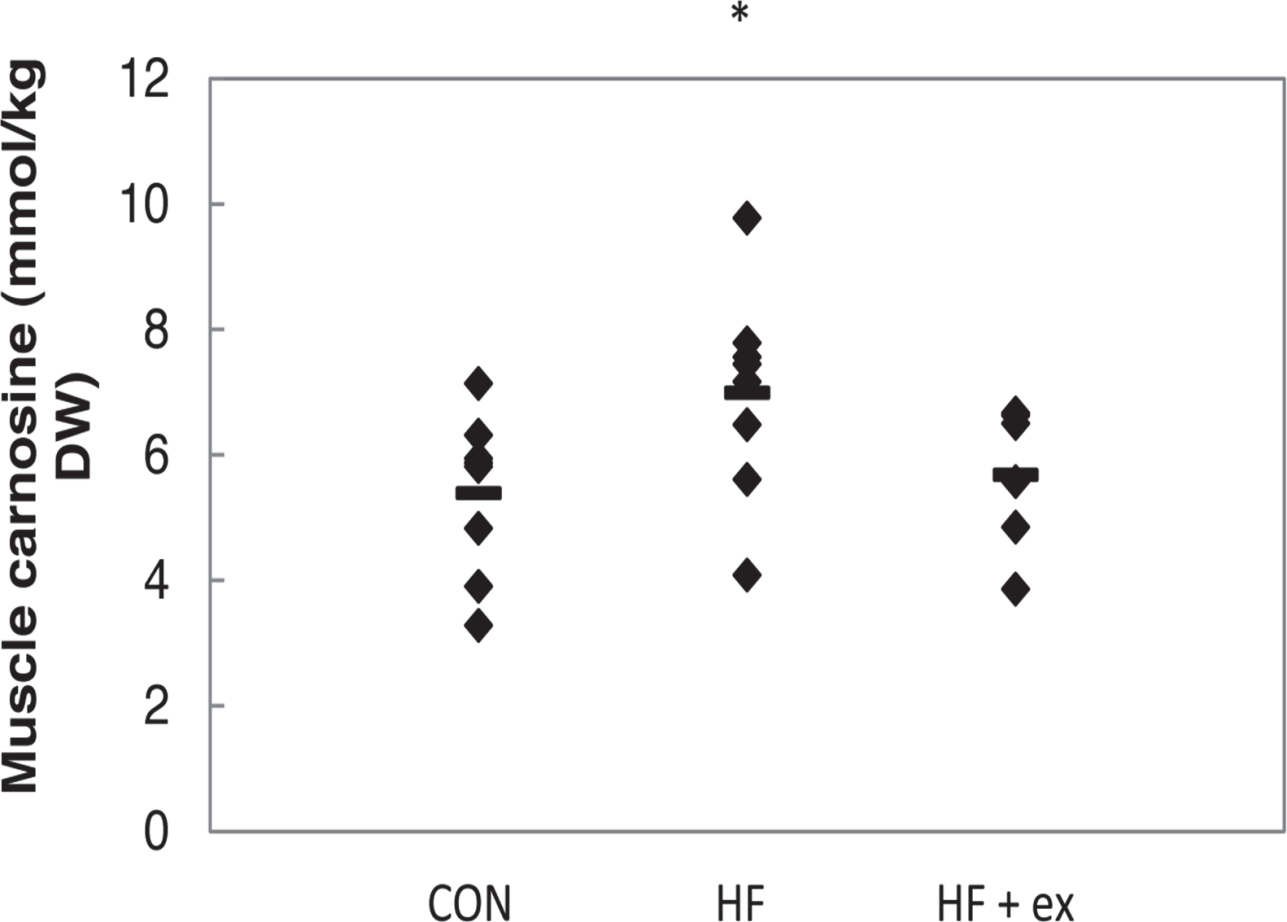
*Rodent study*: The effect of a 70% high-fat diet on muscle carnosine in female mice. Diamonds and stripes represent individual and group values, respectively.* p<0.05 versus CON. Abbreviations: CON: control group, HF: high-fat diet group, HF+ex: high-fat diet+exercise group.

**Rodent study (Table 3).** In the *HF 45%* and *HF 60% study*, neither muscle carnosine, nor anserine, nor the sum (total HCD) was altered by high-fat feeding (for both muscle types GR and GW). Carnosine in GW muscle correlated positively with the AUC of blood glucose during the OGTT (r = 0.542, p<0.05) in the HF 45% study, but not in the HF 60% study or in the GR muscle. In the *CAF study*, muscle anserine and total HCD had tendency to increase in GW by 14% (p = 0.085) and 12% (p = 0.076), respectively. No changes were reported for muscle carnosine and GR and no correlation was found between muscle carnosine and glucose tolerance. In the *HF 70% study*, muscle carnosine significantly increased with 30% (p<0.05) and muscle HCD tended to increase (+21%) in HF compared to CON (p = 0.095) (Fig. 1). Muscle anserine followed a similar pattern, but it was not significant. Daily exercise training (HF 70%+ex) seemed to counteract the high-fat diet-induced increase in muscle carnosine (Fig. 1), anserine and total HCD compared to HF. Muscle carnosine (of the CON and HF mice) correlated positively with the AUC for glucose during the OGTT (r = 0.494, p = 0.052). In addition, muscle carnosine tended to correlate with blood glucose at every time point during the OGTT (fasting: r = 0.478, p = 0.061; after 15 min: r = 0.463, p = 0.071; after 30 min: r = 0.485, p = 0.057; after 60 min: r = 0.514, p<0.05, after 90 min: r = 0.553, p<0.05; after 120 min: r = 0.353, p = 0.18). Anserine did not correlate with glucose intolerance in neither of the rodent studies.

**Table 3:** *Rodent study*: Muscle carnosine, anserine and total histidine-containing dipeptides after the diet-intervention.

		Carnosine		Anserine		HCD	
		mmol/kg DW		mmol/kg DW		mmol/kg DW	
Study	Group	GW	GR	GW	GR	GW	GR
Rat studies							
HF 45%	CON (n = 8)	19.1 ± 4.1	9.2 ± 2.1	34.6 ± 2.4	29.2 ± 7.6	53.7 ± 3.6	38.5 ± 7.8
	HF (n = 7)	18.2 ± 4.0	8.3 ± 1.4	35.7 ± 6.5	29.4 ± 5.4	53.9 ± 9.3	37.7 ± 6.0
HF 60%	CON (n = 9)	13.7 ± 3.6	5.9 ± 2.0	38.3 ± 4.3	25.4 ± 7.0	52.0 ± 4.9	31.3 ± 7.3
	HF (n = 9)	12.8 ± 2.0	4.9 ± 2.0	37.7 ± 4.5	21.8 ± 4.4	50.5 ± 4.7	26.7 ± 4.1
CAF	CON (n = 11)	9.7 ± 1.8	7.1 ± 0.9	30.4 ± 5.1	24.0 ± 4.0	40.1 ± 6.4	31.1 ± 4.9
	CAF (n = 11)	10.5 ± 2.3	7.9 ± 1.3	34.6 ± 5.7^$^	23.3 ± 3.1	45.1 ± 6.2^$^	31.2 ± 3.9
Mouse study							
		**GAS**		**GAS**		**GAS**	
HF 70%	CON (n = 8)	5.4 ± 1.3		11.1 ± 2.4		16.4 ± 3.6	
	HF (n = 8)	7.0 ± 1.7 *		12.9 ± 2.5		19.9 ± 4.1 ^$^	
	HF+ex (n = 6)	5.7 ± 1.1		10.7 ± 1.7 ^$$^		16.4 ± 2.6 ^$$^	

Values are expressed as mean ± SD.

* p< 0.05 HF versus CON,

^$^ p< 0.1 HF vs CON and

^$$^ p<0.1 HF+ex vs HF.

Abbreviations: CON: control group, HF: high-fat group, HF+ex: high-fat+exercise group, DW: dry weight, GW, GR and GAS represent the white, the red and the gastrocnemius as a whole, respectively.

**Fig 2:**
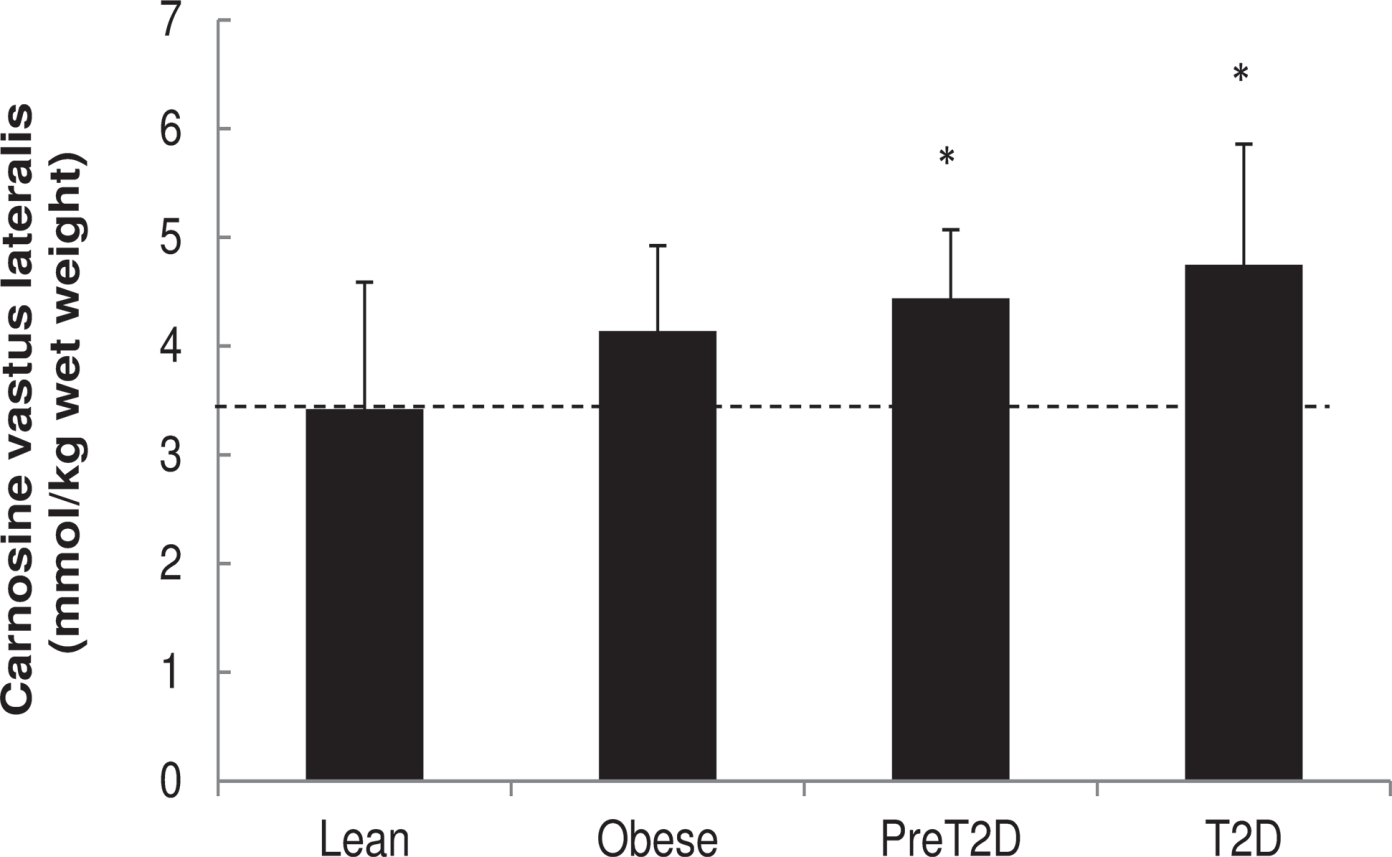
*Human study*: Muscle carnosine content in lean, obese, prediabetic and diabetic type 2 patients. Values are expressed as mean ± SD. * p<0.05 versus lean. ANOVA p-value: 0.038. Abbreviations: preT2D: prediabetic patients, T2D: diabetic type 2 patients.

**Human study.** Muscle carnosine gradually increased with obesity and progressive glucose intolerance. Muscle carnosine of the obese, preT2D and T2D is higher [+21% (p>0.1), +30% (p<0.05) and +39% (p<0.05), respectively], compared to the lean (Fig. 2). Muscle carnosine correlated negatively with the level of medium and high intensity free living ambulatory activity as monitored by accelerometers (r = -0.41, p<0.05). The gene expression analysis of muscle fiber type markers indicated a shift in muscle fiber type composition, with a significant increase in fast-oxidative type 2A fibers (MYH2 mRNA) and a similar trend for fast-glycolytic type 2X fibers (MYH1 mRNA) in muscle of prediabetic and T2D individuals compared to the healthy lean subjects (MYH2 mRNA: obese: 1.1-fold (p>0.1), preT2D: 1.8-fold (p<0.05), T2D: 1.6-fold (p = 0.07) over lean and MYH1 mRNA: obese: 2.2-fold (p>0.1), preT2D: 2.7-fold (p = 0.07), T2D: 2.5-fold (p>0.1) over lean). In addition, expression of MYH1 gene (marker of the fast-glycolytic type 2X muscle fiber content) was positively associated with fasting insulinemia (r = 0.527, p = 0.002). On the other hand, the lack of differences in MYH7 mRNA between the 4 groups indicates that slow-oxidative type I muscle fiber content does not vary with obesity, prediabetes or type 2 diabetes. Levels of muscle carnosine were not associated with the variability in gene expression of muscle fiber type markers (data not shown).

## Discussion

It has been suggested that carnosine levels in diabetic tissues are decreased because carnosine can form adducts with reactive carbonyls species such as HNE and acrolein [14,15], which are abundantly present in diabetic tissues [25]. Indeed, a decrease in carnosine content was reported in retina, kidney and liver of diabetic rodents [6,7,17]. However, literature about carnosine levels in diabetic muscle was contradictive [18–20]. Therefore, we have now examined the effect of different degrees of glucose intolerance on muscle HCD (carnosine and anserine) in both rodents (intervention studies) and men (a cross-sectional study).

The current paper shows that muscle HCD content does not decrease, but instead increases with obesity and progressive glucose intolerance in male individuals. In addition, high-fat diet-induced glucose intolerance was associated with increased muscle HCD in female mice. The observation that muscle tissue responds differently in a diabetic state compared to tissues such as retina, kidney and liver, is probably related to the fact that the enzyme carnosine synthase is considerably more expressed in skeletal muscle compared to the other tissues [31,32], whereby restoration of initially decreased muscle carnosine levels is more easily achieved. Yet, it is possible that a compensation mechanism is activated, ultimately leading to net increased carnosine levels.

In search for an alternative explanation for this unexpected finding, we looked at the known determinants of muscle carnosine content and whether or not glucose intolerance could play an interfering role. In addition to age and sex, muscle fiber type is one of the most important determinants of muscle carnosine content, both in rodents and humans [33–37]. The fast type 2 muscle fibers (both the fast-glycolytic as well as the fast-oxidative muscle fibers) possess almost a double amount of carnosine compared to the oxidative type I muscle fibers [38]. As several studies have shown that obesity and type 2 diabetes are associated with an increased proportion of fast glycolytic type 2 fibers and a reduced percentage of slow type 1 fibers compared with lean healthy individuals, it is plausible to speculate that a shift towards a faster fiber type might contribute to the higher carnosine content in type 2 diabetic muscle.

There are some indications in this paper that support our fiber-type explanation. First, both our rodent and human study demonstrated that aerobic exercise (mice) or physical activity (human), which is known to stimulate or preserve oxidative type 1 fibers [39], counteracted the increase in muscle carnosine. However, it can also be related to the fact that exercise prevents the formation of reactive carbonyl species in skeletal muscle [25], whereby the compensation mechanism to increase muscle carnosine will not be activated. Second, a tendency towards increased gene expression of MYH1 (encoding the protein myosin heavy chain 2X, present in fast-glycolytic type 2 fibers) and MYH2 (encoding the protein myosin heavy chain 2A, present in fast-oxidative type 2 fibers) in individuals with prediabetes or type 2 diabetes suggests a higher proportion of more fast muscle fibers (fast-glycolytic and fast-oxidative) in patients with metabolic disease. Third, the fact that the increase in muscle HCD was only clearly present in mice receiving the extreme 70% high-fat diet, and not in the rats receiving a 45–60% high-fat diet or a cafeteria diet, is perhaps due to the fact that fiber type proportions can change throughout the diet intervention. In the beginning of a high-fat diet intervention, muscle fiber type changes in favor of the oxidative metabolism, including increased type I fibers, in an attempt to remove lipid overload. On the other hand, towards the end of the diet, when glucose intolerance increases, the percentage of fast-glycolytic type 2 fibers increases [40].

But why did Gualano et al. (2012) found a decrease in muscle carnosine in Brazilian T2D patients? Compared to the younger, newly diagnosed drug naïve male T2D subjects in this study, the Brazilian diabetic group is represented by both older (± 60 years) males and females, with longer duration of the disease (± 7 years), receiving oral antidiabetic pharmacotherapy (metformin and/or sulfonylurea). In both studies, patients with micro- and macrovascular complications were excluded. It can be speculated that the compensation mechanism, which is responsible for the initial increase in muscle carnosine, is exhausted by the longer duration of the disease and older age. In other words, it can be expected that carnosine would be largely used to scavenge reactive carbonyl species in the older diabetic muscle, resulting in a net decrease in muscle carnosine content as compared to muscles of younger, drug-naïve newly diagnosed diabetic patients.

Finally, in this work, we demonstrated that muscle HCD content increases with obesity and progressive glucose intolerance in *male* subjects. So, it can be questioned whether this observation would also occur in females. We cannot ignore that the carnosine metabolism is partly gender dependent. Muscle carnosine storage and serum carnosinase (the enzyme that hydrolyses carnosine) is respectively lower and higher in women than in men [41,42]. However, determinants of muscle carnosine storage (such as age and fiber type) and muscle carnosine loading (by beta-alanine supplementation) are gender independent [34,43]. In addition, a 70% high-fat diet increased muscle carnosine in *female* mice. We suggest we can extrapolate this to female humans. Although rodents do not have the enzyme serum carnosinase and although they possess next to carnosine also the methylated variant anserine, it has previously been shown that muscle HCD storage in rodents and humans is quite similar. Both species share the similar determinants of muscle HCD content (age, sex, fiber type, beta-alanine supplementation)[23,33–37].

Both an increase and decrease in muscle carnosine (or anserine) with prediabetes or type 2 diabetes were reported in existing literature [19,20]. However, our results demonstrate a gradual increase in muscle HCD with progressive glucose intolerance in male individuals. In addition, high-fat diet-induced glucose intolerance is associated with increased muscle HCD in female mice. We underscore the importance of future studies aiming to better understand the metabolic pathways and the (patho)physiological significance of HCD in muscle in particular and in the body in general, in order to optimally design therapeutic strategies interfering with the carnosine system in metabolic diseases.
